# Protective Effects of BDNF against C-Reactive Protein-Induced Inflammation in Women

**DOI:** 10.1155/2015/516783

**Published:** 2015-05-26

**Authors:** Nicole Noren Hooten, Ngozi Ejiogu, Alan B. Zonderman, Michele K. Evans

**Affiliations:** Laboratory of Epidemiology and Population Sciences, National Institute on Aging, National Institutes of Health, 251 Bayview Boulevard, Baltimore, MD 21224, USA

## Abstract

*Background*. Since high sensitivity C-reactive protein (hsCRP) is predictive of cardiovascular events, it is important to examine the relationship between hsCRP and other inflammatory and oxidative stress markers linked to cardiovascular disease (CVD) etiology. Previously, we reported that hsCRP induces the oxidative stress adduct 8-oxo-7,8-dihydro-2′deoxyguanosine (8-oxodG) and that these markers are significantly associated in women. Recent data indicates that brain-derived neurotrophic factor (BDNF) may have a role in CVD. *Methods and Results*. We examined BDNF levels in 3 groups of women that were age- and race-matched with low (<3 mg/L), mid (>3–20 mg/L), and high (>20 mg/L) hsCRP (*n* = 39 per group) and found a significant association between hsCRP, BDNF, and 8-oxodG. In African American females with high hsCRP, increases in BDNF were associated with decreased serum 8-oxodG. This was not the case in white women where high hsCRP was associated with high levels of BDNF and high levels of 8-oxodG. BDNF treatment of cells reduced CRP levels and inhibited CRP-induced DNA damage. *Conclusion*. We discovered an important relationship between hsCRP, 8-oxodG, and BDNF in women at hsCRP levels >3 mg/L. These data suggest that BDNF may have a protective role in counteracting the inflammatory effects of hsCRP.

## 1. Introduction

Continued efforts to enhance our understanding of cardiovascular disease (CVD) in women have strengthened risk assessment, diagnosis, and treatment of this disease; however, CVD remains the primary cause of death of women over the age 25 [1]. Furthermore, well-known racial disparities exist for CVD in the United States as African American (AA) women have higher rates of CVD and hypertension than white women [2]. Recent evidence suggests that levels of the CVD risk factor high sensitivity C-reactive protein (hsCRP) differ among AAs and whites and are elevated in females versus males [1, 3]. High serum levels of hsCRP pose a significant risk for future CVD events. Thus, continued assessment of this inflammatory marker is an important step towards prevention and diagnosis of CVD [4]. It is also important to analyze the relationship of hsCRP to other CVD risk factors and inflammatory markers.

Brain-derived neurotrophic factor (BDNF) is a well-established neurotrophic factor and studies using mouse models have demonstrated that BDNF and its receptor tropomyosin-related kinase B (TrkB) also have critical roles in the development of the cardiovascular system [5, 6]. BDNF-deficient mice develop hypocontractility of the heart that leads to early postnatal death [7]. BDNF has been shown to promote neoangiogenesis and enhance blood flow following mouse ischemic injury. It is also upregulated by the central nervous system in order to protect against cardiac remodeling after myocardial infarction [8, 9]. These data suggest that BDNF plays a role not only in mouse cardiovascular development but also in CVD and related pathologies.

The role of BDNF in human CVD is less well understood. Low levels of BDNF have been reported in patients with acute coronary syndrome and in association with increased incidence of coronary events in Chinese patients with angina pectoris [10, 11]. In elderly participants in the Baltimore Longitudinal Study of Aging, BDNF was positively associated with several CVD risk factors including body mass index, diastolic blood pressure, and metabolic syndrome and these associations varied by sex [12]. In one study, BDNF staining was observed in atherosclerotic coronary arteries but not in nonatherosclerotic coronary arteries [13]. BDNF may lead to plaque instability in atherosclerotic plaques through its ability to induce oxidative stress and promote the generation of superoxide radicals [13, 14]. BDNF has also been shown to induce oxidative stress by activating the NAD(P)H oxidase system in coronary vasculature [13]. However, because of the role of BDNF in the survival and development of endothelial cells, BDNF may have dual roles in the cardiovascular system [13]. These studies suggest a potential association of BDNF with human CVD; however, how BDNF may contribute to CVD is not well understood and the relationship between BDNF and the CVD risk factor hsCRP has not been investigated. Furthermore, little is known about how BDNF levels are influenced by race.

Previously, we reported a significant relationship between the oxidative stress marker 8-oxodG and hsCRP in a subcohort of women from the Healthy Aging in Neighborhoods of Diversity across the Life Span (HANDLS) study—a longitudinal, epidemiological study of health disparities based in Baltimore, MD [15]. We found that serum levels of the DNA base lesion 8-oxodG were independently associated with systolic pressure and pulse pressure, both markers of vascular health. CRP induces reactive oxygen species and DNA base lesions, suggesting that CRP may contribute to CVD by increasing oxidative stress [15]. The aim of this study was to examine the possible interrelationship between BDNF and hsCRP in the setting of CVD risk in women of different races.

## 2. Materials and Methods

### 2.1. Study Design

Previously, we performed a nested-cohort study of women with low (<3 mg/L), mid (>3–20 mg/L), and high (>20 mg/L) hsCRP levels [15]. Each group (*n* = 39) was matched on age and race and are part of the Healthy Aging in Neighborhoods of Diversity across the Life Span (HANDLS) study of the National Institute on Aging Intramural Research Program (NIA IRP) [16]. Clinical characteristics of the cohort are included in Table 1. The study is approved by the Institutional Review Board of the National Institute of Environmental Health Sciences, NIH, and the study protocol conforms to the Ethical Guidelines of the 1975 Declaration of Helsinki. HANDLS is an interdisciplinary, epidemiologic study on health disparities and aging in a cohort of urban adults (ages 30–64) in Baltimore city. Women were chosen for this subcohort if they gave written consent to store serum, had available serum for examination, and had completed the HANDLS baseline assessment.

We matched three groups of women (39 per group) on age and race into groups based on hsCRP level defined in our previous cohort study [15]. These women had low (<3 mg/L), mid (>3–20 mg/L), or high (>20 mg/L) hsCRP levels. Race and sex were both self-reported from participants. Eighty-six women in the total HANDLS study cohort had hsCRP values >20 mg/L. The cohort contains premenopausal (*n* = 13 in low group, *n* = 10 in mid group, and *n* = 11 in high group) and postmenopausal (*n* = 24 in low group, *n* = 28 in mid group, and *n* = 24 in high group) women. Group sizes were determined by a power analysis, which showed that 37 women per group provided sufficient power to detect differences at least as large as one-third of a standard deviation using *p* < 0.05.

### 2.2. Physical Measurements, Laboratory, and 8-oxodG Assays

Blood pressure was taken in both arms and averaged for assessments in both arms while seated after a five-minute rest. Body mass index (weight [kg]/height [m]^2^) was computed from measured height and weight. Clinical conditions were recorded based on a structured medical history interview and a physical examination. Fasting blood samples were obtained and the serum was assayed by Quest Diagnostics (Nichols Institute, Chantilly, VA) or stored at −80°C. Fasting glucose, insulin, cholesterol, triglycerides, LDL, HDL, creatinine, LDH, and hsCRP were measured at Quest Diagnostics. BDNF and other cytokine and inflammatory markers were measured in serum using Searchlight protein arrays from Aushon Biosystems (Billerica, MA) [15]. Serum 8-oxodG ELISA assays were performed blindly previously [15] according to the manufacturer's instructions (Genox, Inc., Gaithersburg, MD).

### 2.3. Cell Lines and Reagents

Human umbilical endothelial cells (HUVEC; Lonza) were maintained in EBM-2 media supplemented with EGM-2 SingleQuots (Lonza). Human cardiac microvascular endothelial cells (HMVEC-C; Lonza) were maintained in EBM-2 media supplemented with EGM-2V SingleQuots (Lonza). HepG2 hepatocarcinoma cells were purchased from ATCC and grown in modified eagle's medium (MEM) supplemented with 10% FBS, L-glutamine, and sodium pyruvate. Human recombinant brain-derived neurotrophic factor (BDNF) was purchased from Sigma-Aldrich and highly purified C-reactive protein (CRP), sodium azide and endotoxin free, was obtained from TriChem Resources Inc.

### 2.4. Quantification of mRNA and Protein Levels

HepG2 cells were incubated in serum-free media and HUVECs were incubated in a 1 : 10 dilution of growth media to serum-free media overnight with or without 1 or 10 ng/mL BDNF and the next day cells were scraped and the cell pellet was split to examine both protein and mRNA levels from the same sample. Total RNA was isolated using TRIzol according to the manufacturer's instructions. RNA was quantified using NanoDrop ND-1000 Spectrophotometer and equal amounts were reverse-transcribed using random hexamers and SSII reverse transcriptase (Invitrogen). Real-time RT-PCR was performed using gene-specific primer pairs and SYBR Green PCR master mix (Applied Biosystems) on an Applied Biosystems 7500 Real-Time PCR machine. The indicated primers used were CRP forward 5′-AGACATGTCGAGGAAGGCTTTT and reverse 5′-TCGAGGACAGTTCCGTGTAGAA and GAPDH forward 5′-TGCACCACCAACTGCTTAGC and reverse 5′-GGCATGGACTGTGGTCATGAG.

For protein analysis, cells were lysed directly in 2X Laemmli sample buffer, boiled, and analyzed using SDS-PAGE. Immunoblots were probed with anti-CRP antibodies (Millipore), anti-TrkB antibodies (Cell Signaling), anti-BDNF (Abcam), anti-APE-1 (Novus Biologicals), and anti-actin antibodies (Santa Cruz Biotechnologies) as a protein loading control.

### 2.5. 8-oxoG Staining

HMVEC-Cs were untreated or pretreated for 18 hrs with 10 ng/mL BDNF in growth media and then treated with either 25 *μ*M menadione (Sigma-Aldrich), 25 *μ*g/mL CRP, 10 ng/mL BDNF, or BDNF and CRP for 30 min in serum-free EBM-2 media. 8-oxoG staining was performed as previously described [17]. Fluorescent images of 8-oxoG and 4′,6-diamidino-2-phenylindole DAPI were taken on a Zeiss Observer D1 microscope with an AxioCam1Cc1 camera at a set exposure time, and the fluorescence intensity of 8-oxoG-stained nuclei was quantified from duplicate coverslips using AxioVision Rel. 4.7 software. The histogram represents the normalized average from four experiments.

### 2.6. Statistical Analyses

We used mixed-model regressions to examine effects on 8-oxodG after adjusting for covariates. We included interactions in regression analyses only after they were significant following a backward elimination procedure, a standard statistical method used to select variables and optimize regression models by sequentially excluding nonsignificant effects [18]. We applied a log⁡10 transform to hsCRP because the distribution was skewed. We used R [19] to perform all analyses and to draw graphs. We set statistical significance as *p* < 0.05.

## 3. Results

### 3.1. Association of 8-oxodG, BDNF, and hsCRP Differs by Race

Here we have examined serum BDNF levels in a subcohort of women from the HANDLS study with low (<3 mg/L), mid (>3–20 mg/L), or high hsCRP levels (>20 mg/L). Each group contained 39 women and the groups were matched on both age (mean age, 49.7 + 8.1 years) and race (19 whites and 20 African Americans). Clinical characteristics of the cohort are described in Table 1. We wanted to examine the relationship of CVD risk factors and oxidative stress and inflammatory markers over the full range of hsCRP levels, as data from the Women's Health Study has shown that women with high levels (≥10 mg/L) of hsCRP have a high risk and women with very high levels of hsCRP (>20 mg/L) were at the very highest risk for future cardiovascular events [20–22]. The mean BDNF levels were 8.37, 8.57, and 38.04 ng/mL for the low, mid, and high level hsCRP groups, respectively (Table 1). Compared to the low hsCRP group, BDNF levels were significantly higher in the mid CRP group (*p* ≤ 0.0001), but not statistically significant in the high hsCRP group.

We examined the association of BDNF with serum 8-oxodG adjusting for race and poverty status and found a significant, though small, interaction between BDNF and race. We found no significant associations with poverty status and retained the nonsignificant main effects for BDNF and race in the presence of their significant two-way interaction (BDNF × race: *b* = 6.1 × 10^−7^; 95% CI = 1.1 × 10^−7^, 1.1 × 10^−6^; *p* = 0.017).

**Figure 1:**
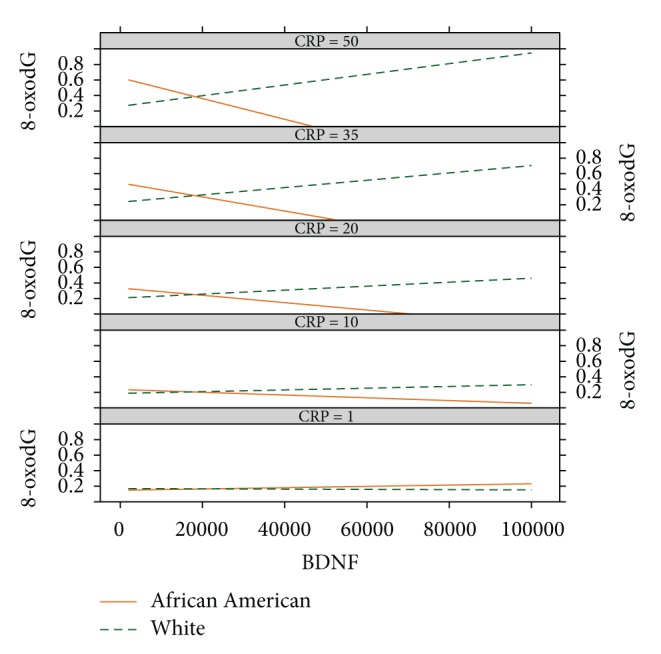
Association of 8-oxodG on BDNF at selected levels of hsCRP in African Americans (AA) and whites. For the graphs, each box corresponds to different levels of hsCRP. BDNF levels are indicated on the *x*-axis and 8-oxodG levels are shown on the *y*-axis. At low levels of hsCRP, 8-oxodG levels are identical between AAs and whites at all levels of BDNF. However, with increasing levels of hsCRP and at high levels of BDNF, white women have elevated levels of 8-oxodG compared with AA women.

In an attempt to account for this interaction, we examined inflammatory markers hsCRP, IL-18, TNF-*α*, and RAGE to determine whether they moderated the association of BDNF and race with 8-oxodG. Although there were no significant main effects, there were significant interactions between BDNF × race (1.4 × 10^−6^; 95% CI = 2.3 × 10^−6^, 5.4 × 10^−7^; *p* = 0.002) and between BDNF × race × log⁡(hsCRP) (−4.5 × 10^−7^; −8.5 × 10^−8^, −8.1 × 10^−7^; *p* = 0.016) in a model adjusted for BDNF, race, and log⁡(hsCRP) variables. At low levels of hsCRP (hsCRP = 1 and 10), the relationship between 8-oxodG (*y*-axis) and BDNF (*x*-axis) is identical in whites (dashed green line) and AAs (solid orange line) (Figure 1). However, at increasing levels of hsCRP, the relationship between 8-oxodG and BDNF diverges. In whites, 8-oxodG increases as BDNF increases. In AAs, 8-oxodG decreases as BDNF increases, suggesting that in AA women with elevated levels of hsCRP, BDNF may protect against CRP-induced DNA damage more efficiently than in white women.

### 3.2. Relationship between 8-oxodG and BDNF with Pulse Pressure Differs by Race

**Figure 2:**
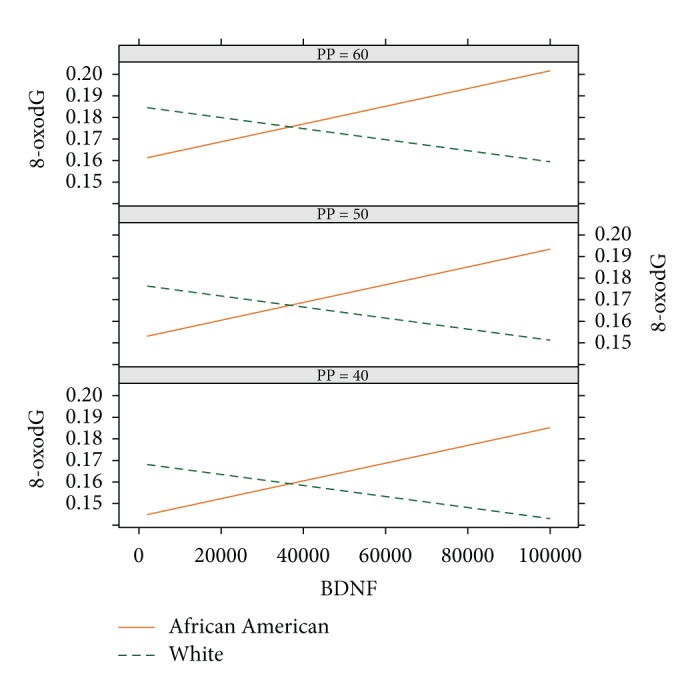
Association of 8-oxodG on BDNF at selected levels of pulse pressure in African Americans (AA) and whites. For the graphs, each box indicates different levels of pulse pressure (PP). BDNF is indicated on the *x*-axis and 8-oxodG is shown on the *y*-axis. At all levels of pulse pressure and at high levels of BDNF, AA women have high levels of 8-oxodG whereas white women have low levels of 8-oxodG.

As a follow-up to our previous finding of an association between pulse pressure and 8-oxodG, we also examined the association of BDNF, race, and pulse pressure on 8-oxodG and found a significant association with 8-oxodG for race (*b* = −0.025; 95% CI = −0.006, −0.043; *p* = 0.008), pulse pressure (*b* = 8.2 × 10^−4^; 95% CI = 3.5 × 10^−4^, 1.3 × 10^−3^; *p* = 0.001), and BDNF × race (*b* = 6.8 × 10^−7^; 95% CI = 1.7 × 10^−7^, 1.2 × 10^−6^; *p* = 0.009). At all levels of pulse pressure (PP), high levels of BDNF (*x*-axis) in AAs (solid orange line) are associated with high levels of 8-oxodG (*y*-axis) whereas high levels of BDNF in whites (dashed green line) are associated with low levels of 8-oxodG (Figure 2). The overall levels of 8-oxodG increases as pulse pressure increases [15].

We tested a variety of moderators in an attempt to explain these associations. However, cigarette smoking, illicit substance use, diabetes mellitus, symptoms of depression, or putative genetic markers for BDNF (rs6265 [Val66MET], rs1519480) and CRP (rs3093080, rs3093066, rs3093062, rs3093059, rs3093058, and rs3091244) were not associated significantly with 8-oxodG. None of these measures eliminated the significant associations of BDNF and pulse pressure with 8-oxodG.

Given that our cohort is women and that estrogen has also been reported to be associated with BDNF [23], we also included menopause in the models. Menopause was not associated significantly with 8-oxodG and did not change the relationship between BDNF, pulse pressure with 8-oxodG, or the relationship between BDNF, race, and CRP.

### 3.3. BDNF Reduces CRP Levels and Inhibits CRP-Induced DNA Base Lesions

**Figure 3:**
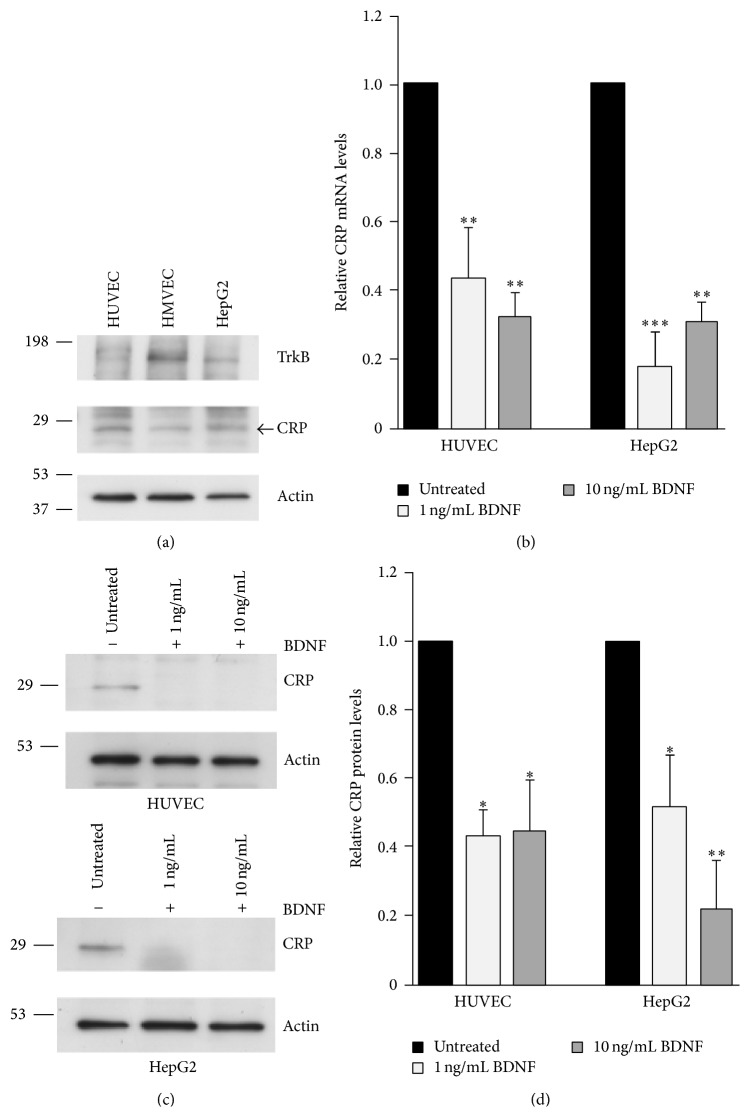
BDNF reduces CRP levels in cells. (a) HUVECs, HMVEC-C, or HepG2 cells were lysed, separated by SDS-PAGE and probed with anti-TrkB, anti-CRP, and anti-actin antibodies as a loading control. (b) HUVECs were untreated or treated with the indicated concentrations of BDNF for 18 hrs and CRP mRNA levels were assessed by RT-qPCR and normalized to GAPDH. (c) Cells treated as in (b) were lysed and probed with anti-CRP and anti-actin antibodies as a protein loading control. (d) CRP protein levels were quantified from immunoblots and normalized to actin. The histograms represent the normalized mean + SEM from 3 independent experiments. ^*∗*^ *p* < 0.05, ^*∗∗*^ *p* < 0.01, and ^*∗∗∗*^ *p* < 0.001 using one-way ANOVA and Tukey's post hoc test.

**Figure 4:**
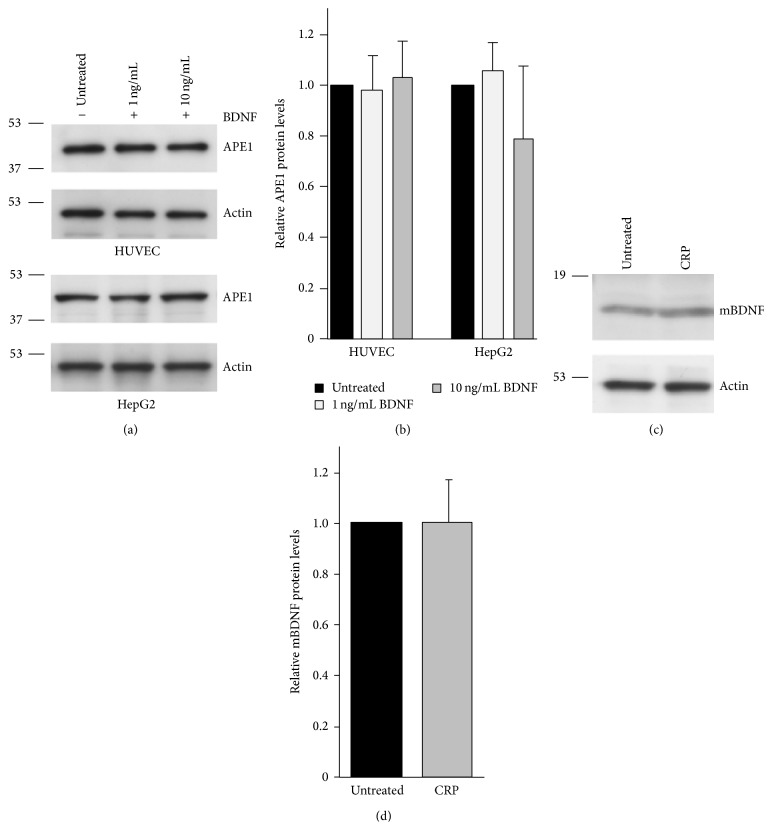
CRP or BDNF treatment does not affect BDNF or APE1 levels, respectively. (a) HUVECs or HepG2 cells were untreated or treated with the indicated concentrations of BDNF for 18 hrs. Cells were lysed, separated by SDS-PAGE, and probed with anti-APE1 and anti-actin antibodies. (b) APE1 protein levels were quantified from immunoblots and normalized to actin. The histograms represent the normalized mean + SEM (*n* = 5 for HepG2 and *n* = 3 for HUVEC). (c) HUVECs were treated for 18 hrs with 25 *μ*g/mL CRP. Cells were lysed, separated by SDS-PAGE, and probed with anti-BDNF and anti-actin antibodies as a loading control. Mature BDNF (mBDNF) is indicated. (d) BDNF protein levels were quantified from immunoblots and normalized to actin (*n* = 5).

Given the significant relationship between BDNF, hsCRP, and 8-oxodG in women, we investigated whether BDNF counteracts or potentiates oxidative stress induced by CRP. Initially, we examined several cell lines for expression of the BDNF receptor, TrkB. TrkB is expressed in human umbilical vein cells (HUVEC), human cardiac microvascular endothelial cells (HMVEC-C), and the hepatocarcinoma cell line HepG2 (Figure 3(a)). Consistent with the vascular expression of TrkB in the developing heart and the adult mouse, the highest expression of TrkB was observed in the HMVEC-C cells [6]. CRP is expressed in these cell lines as well, with the highest expression in HUVECs and HepG2 cells, which is consistent with hepatocytes being a major source of circulating CRP (Figure 3(a)). Therefore, we examined whether BDNF influenced levels of CRP in these cells. Treatment with BDNF at two different doses significantly decreased the mRNA and protein levels of endogenous CRP in both cell lines (Figures 3(b)–3(d)). In neurons, BDNF was recently reported to upregulate protein levels of the DNA repair protein, APE1 [24]. However, BDNF does not affect levels of APE1 in endothelial or hepatocellular cells (Figures 4(a) and 4(b)). In addition, we also found that CRP treatment did not significantly affect BDNF levels in endothelial cells (Figures 4(c) and 4(d)).

**Figure 5:**
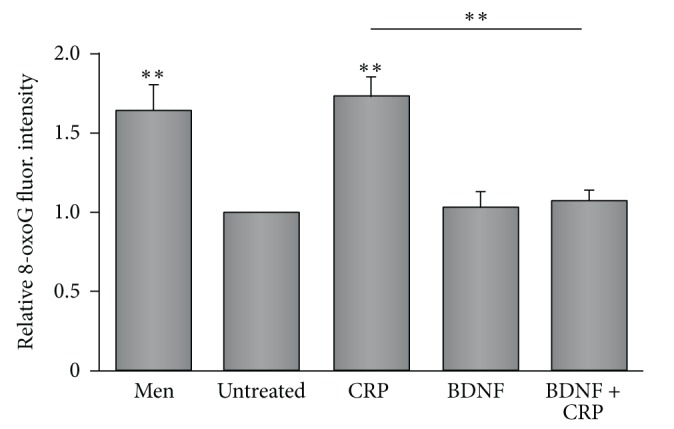
BDNF inhibits CRP-induced DNA damage. HMVEC-Cs untreated or treated with 25 *μ*M menadione (Men), 25 *μ*g/mL CRP (CRP), 10 ng/mL BDNF (BDNF), or BDNF and CRP (BDNF + CRP) and then fixed and stained with anti-8-oxoG antibodies. Fluorescent intensity for 8-oxoG stained nuclei was quantified and the histogram represents the normalized mean + SEM from 4 experiments. ^*∗∗*^ *p* < 0.01 using one-way ANOVA and Tukey's post hoc test.

Previously, we reported that CRP induces ROS and 8-oxoG lesions in endothelial cells [15]. In order to examine whether BDNF can affect CRP induced oxidative DNA damage, we treated HMVEC-C cells with highly purified recombinant CRP in the presence of recombinant BDNF. We chose HMVEC-C since these cells had the highest levels of TrkB expression (Figure 3(a)). As previously reported for HUVECs, hsCRP increased levels of 8-oxoG in HMVEC-Cs [15] (Figure 5). In addition, menadione (MEN) was used as a positive control since this agent is well known to induce 8-oxoG lesions (Figure 5). Similar levels of 8-oxodG lesions were observed between untreated and BDNF treated cells, indicating that BDNF does not induce 8-oxoG (Figure 5; 4th bar in histogram). However, BDNF inhibited CRP induced 8-oxoG lesions (Figure 5; comparing 3rd to 5th bars in histogram). Since BDNF decreases CRP expression and blocks CRP-induced oxidative DNA damage, these data suggest that BDNF plays a protective role in inhibiting the oxidative damage effects of CRP on vascular cells.

## 4. Discussion

Here, we have investigated the relationship of BDNF with hsCRP and a marker of oxidative stress. In women at high risk for cardiovascular events, we found a significant relationship between BDNF and 8-oxodG that was different in AA women than in white women. In the high hsCRP group (hsCRP ≥ 20 mg/L), AA women had serum levels of BDNF that increased with decreasing levels of serum 8-oxodG. White women in the high hsCRP group (hsCRP ≥ 20 mg/L) had serum levels of BDNF that increased with increasing levels of serum 8-oxodG. We further investigated the relationship of BDNF, hsCRP, and 8-oxodG* in vitro* using cell culture models. We found that BDNF can reduce CRP mRNA and protein levels. BDNF also inhibited CRP-induced oxidative DNA damage. These data suggest that BDNF may have a protective role in the cardiovascular system.

The idea that BDNF may have a cardioprotective role is consistent with other reports. For example, in mice, exercise increases BDNF levels and exercise is well known to be beneficial to both the cardiovascular system and the brain [25]. Other modulators of cellular stress such as dietary energy restriction, shear stress, and hypoxia also upregulate levels of BDNF [25–28]. Therefore, BDNF is postulated to be part of an adaptive response to stress that helps protect the brain and potentially the heart [25–28]. Here we found an association between the oxidative stress marker 8-oxodG, BDNF, and hsCRP. It is interesting to speculate that BDNF may be upregulated in response to the increasing levels of circulating hsCRP in these women. Consistent with this idea, high levels of BDNF were associated with cardiopulmonary fitness in patients with coronary artery disease [29].

Although these reports suggest that BDNF may have a protective role in the cardiovascular system, the role of BDNF in CVD is still very complex and not completely understood. For instance, low plasma levels of BDNF were found to be an independent predictor of a major coronary event in a Chinese cohort of patients with angina pectoris [10]. In a Danish study, low levels of plasma BDNF were associated with higher mortality [30]. Interestingly, this relationship was only observed in elderly women and not men. However, the opposite association has also been observed. In one study, plasma BDNF levels were positively correlated with several CVD risk factors and metabolic syndrome in white elderly subjects (mean age ~70 yrs) [12]. Furthermore, in mice, BDNF has been shown to have opposing roles following myocardial infarction [9, 31]. In one report, BDNF had a protective role in promoting cardiac remodeling after myocardial infarction [9]. In another report, BDNF negatively affected survival after myocardial infarction [31]. Therefore, these data suggest that we have a lot to learn about the precise role of BDNF with relation to CVD.

We found that at incrementally higher levels of hsCRP the relationship between 8-oxodG and BDNF differs by race. It is possible that hsCRP confers different relative risk in each group. In AA women with high levels of hsCRP, BDNF may help moderate oxidative stress induced by increased hsCRP [32]. It is possible that in white women with high levels of hsCRP that BDNF may not work as efficiently to help decrease oxidative stress levels or to counteract the effects of hsCRP. Why there are different racial effects remains to be determined. This subcohort was not designed to evaluate the role of poverty or other important social determinates of health (behavior, societal psychogenic stresses like perceived discrimination, or education). There may be an underlying and unmeasured process that enhances or modulates biological processes resulting in adverse physiologic outcomes. We found that BDNF inhibits CRP-induced 8-oxoG lesions and it has also been reported that BDNF enhances DNA repair [24]. It may be that BDNF affects DNA repair capacity or DNA damage induction levels differently between whites and AAs with high hsCRP. Additionally, the role of BDNF and hsCRP-induced inflammation, such as the prothrombotic activity of hsCRP, needs to be investigated [32].

Previously it has been shown that the BDNF_val/met_ polymorphism was associated with unstable angina in a Chinese cohort [33]. Therefore, we examined genotyping data from our cohort. Polymorphisms in BDNF (including Val66Met) or CRP, however, did not explain the relationships between BDNF, hsCRP, and 8-oxodG. This may be due to the smaller sample size of our study. Larger sample sizes are required to detect variations in BDNF/hsCRP levels explained by different genotypes. Moreover, the BDNF_val/met_ has a smaller minor allele frequency in AA (0.01) and whites (0.21) compared to the Chinese population (0.49).

Very little has been reported at all about circulating levels of BDNF in AAs regardless of sex. A recent report examining BDNF and cognitive decline from the Health ABC study suggests that in older women (mean age 74.9 years) BDNF levels differ by race [34]. Therefore, it will be important in future studies to further examine in both whites and AAs the role that BDNF plays in metabolism and cardiovascular health. As AA females have disproportionate CVD incidence and mortality rates, it is even more important to gain more knowledge about the differences between markers in whites and AAs and what they mean. This may prove important for evaluating the predictive value of risk and diagnostic factors for CVD in AAs versus whites and for determining therapeutic efficacy for treatments for CVD.

## 5. Conclusions

Our findings suggest that there is an important and perhaps clinically relevant relationship between BDNF and CRP and that this relationship may modulate the amount of oxidative DNA damage and/or oxidative stress present in the vasculature. BDNF may attenuate the inflammatory or oxidative stress associated with high levels of CRP, which are directly associated with cardiovascular risk. This may be particularly important in women who have higher hsCRP levels especially African American women.

## Figures and Tables

**Table 1:** Clinical characteristics of cohort with separate comparisons between middle and high hsCRP groups with the low hsCRP group.

	hsCRP groups (*n* = 39 per group)		
	Low	Mid	High
	(≤3 mg/L)	(>3–20 mg/L)	(>20 mg/L)
	Mean	Mean	Mean
Age (y)	49.8 (8.8)	49.8 (8.8)	49.8 (8.8)
hsCRP^1^	−0.46 (1.0)	1.87 (0.5)^*∗∗*^	3.4 (0.4)^*∗∗*^
8-oxodG	0.16 (0.04)	0.17 (0.04)	0.18 (0.04)^*∗∗*^
BMI	26.8 (5.1)	34.74 (9)^*∗∗*^	39.6 (12)^*∗∗*^
Weight^2^	71.7 (14.6)	93.2 (23.7)^*∗∗*^	103.0 (33)^*∗∗*^
Waist size^3^	92.4 (13.5)	106.9 (17.4)^*∗∗*^	115 (23.4)^*∗∗*^
Hip size^3^	102.7 (13.3)	115.8 (17.6)^*∗∗*^	124.4 (21)^*∗∗*^
Waist-hip ratio	0.9 (0.08)	0.92 (0.07)	0.92 (0.08)
Diastolic BP^4^	71.5 (11.7)	75.1 (11.3)	69.9 (8.8)
Systolic BP^4^	118.6 (18.2)	126.4 (18.5)	125.5 (20.4)^#^
Pulse pressure	47.1 (13.0)	51.3 (14.4)	55.6 (15.8)^*∗*^
Total cholesterol^5^	187.1 (34.9)	196.1 (43.4)	182.2 (45.9)
LDL^5^	104.1 (34.7)	115.5 (37.4)	108.9 (43.8)
HDL^5^	60.3 (16.1)	52.5 (14)^*∗*^	47.7 (13.3)^*∗∗*^
Triglycerides^5^	114.2 (81.2)	140 (74.6)^#^	127.8 (61.3)
Creatinine^5^	0.96 (0.9)	0.87 (0.19)	0.84 (0.2)
Glucose^5^	94.2 (10.1)	100.9 (17.2)	150.6 (81)^*∗∗*^
Insulin^6^	10.3 (6.9)	12.3 (8.8)	17.2 (12.7)^*∗*^
LDH^7^	160.4 (32.3)	178.2 (36.3)	163.1 (34.8)
eGFR	86.3 (18.5)	85.9 (21.5)	90.9 (25.4)
BDNF^8^	8.37 (22.5)	8.57 (18.6)^*∗∗∗*^	38.04 (27.2)^#^

Means and standard deviations () are indicated.

^1^log_10_⁡(hsCRP) in mg/L.

^2^kg.

^3^cm.

^4^mm Hg.

^5^mg/dL.

^6^ *μ*IU/mL.

^7^U/L.

^8^ng/mL.

^#^ *p* = 0.08; ^*∗*^ *p* < 0.05;  ^*∗∗*^ *p* < 0.01; ^*∗∗∗*^ *p* < 0.001.
